# Health information technology implementation - impacts and policy considerations: a comparison between Israel and Portugal

**DOI:** 10.1186/s13584-015-0040-9

**Published:** 2015-08-13

**Authors:** Gabriel Catan, Rita Espanha, Rita Veloso Mendes, Orly Toren, David Chinitz

**Affiliations:** Braun School of Public Health, Faculty of Medicine, Hebrew University, Jerusalem, Israel; ISCTE-IUL (University Institute of Lisbon), Lisbon, Portugal; CIES-IUL and Escola Nacional de Saúde Pública, Universidade Nova de Lisboa, Lisbon, Portugal; Hadassah Medical Organization, Jerusalem, Israel

**Keywords:** ICT, eHealth, mHealth, Health policies, Patient empowerment

## Abstract

The use of Information and Communications Technology (ICT) in health systems is increasing worldwide. While it is assumed that ICT holds great potential to make health services more efficient and grant patients more empowerment, research on these trends is at an early stage. Building on a study of the impact of ICT on physicians and patients in Israel, a Short Term Scientific Mission (STSM) sponsored by COST Net in conjunction with CIES/ISCTE IUL (Portugal) facilitated a comparison of ICT in health in Israel and Portugal. The comparison focused on patient empowerment, physician behavior and the role of government in implementing ICT.

The research in both countries was qualitative in nature. In-depth interviews with the Ministry of Health (MOH), the private sector, patients associations, health plans and researchers were used to collect data. Purposeful sampling was used to select respondents, and secondary sources were used for triangulation.

The findings indicate that respondents in both countries feel that patient empowerment has indeed been furthered by introduction of ICT. Regarding physicians, in both countries ICT is seen as providing more information that can be used in medical decision making. Increased access of patients to web-based medical information can strengthen the role of patients in decision making and improve the physician-patient relationship, but also shift the latter in ways that may require adjustments in physician orientation. Physician uptake of ICT in both countries involves overcoming certain barriers, such as resistance to change. At the national level, important differences were found between the two countries. While in Israel, ICT was promoted and adopted by the *meso* level of the health system, in particular the health plans and government intervention can be found in a later stage, in Portugal the government was the main developer and national strategies were built from the beginning. These two approaches present different advantages and disadvantages. Government involvement in earlier stages could provide benefit in terms of interoperability of systems between different healthcare organizations. However, innovation could be slowed down due to government bureaucracy or lack of leadership.

The work provides information in order to understand and improve ICT services. Additionally, it provides input regarding impact of ICT on the physician/patient relationship and national policies in the area.

## Background

### The increasing utilization of information & communication technology

Information & Communication Technology (ICT) utilization has been increasing at a tremendous pace over the last years, and in particular, the use of mobile devices. Today there are more than 7 billion mobile-phone subscriptions around the world, reaching a global penetration of 96 % in 2014. Additionally, the number of Internet users has more than doubled between 2005 and 2014 reaching almost 3 billion [1]. Moreover, Internet, and in particular, mobile phones enjoy more capabilities and new infrastructures that enable larger scale communication. The rapid development of ICT offers great opportunities for many sectors to improve efficiency and reduce costs, but at the same time it poses new challenges. While the population can access more information in real-time, legal issues, such as privacy and security, and social problems, such as the digital divide, need to be addressed as well.

### The utilization of ICT in healthcare

The dynamic environment of increasing competition, limited resources, sociological, economic and political changes, forces organizations to revitalize and improve their performance. Innovation can improve economic performance. Today, healthcare systems are facing many challenges such as the shortage of medical human resources, the increasing costs both in primary care and hospitals, the dynamics of the populations, and the increasing prevalence of chronic diseases and non-communicable diseases [2, 3]. In this environment ICT has been seen as making a potential contribution: “The increased use of technology can help reduce health care costs by improving efficiencies in the health care system and promoting prevention through behavior change communication. It also has the potential to advance clinical care and public health services by facilitating health professional practice and communication and reducing health disparities by applying new approaches to improve the health of isolated populations” [3].

Effects of the implementation of ICT can be studied at three levels: the micro level (users and physicians), the *meso* level (the healthcare organization) and the macro level (the government) [4]. In the first level, patients might have more capability and access to information, thus increasing their empowerment and their voice, providing more choices and better decision making. However, the patient-physician relationship may be affected, and the physicians sometimes may be reluctant to changes in the way that technology is moving. At the organization level, implementation of these tools can affect costs and improve the administration of clinical and financial data. At the macro-governmental level, eHealth can be regulated so as to improve the efficiency and equity of health care delivery systems, but also pose difficult regulatory legal challenges related to privacy and proprietary interests in local eHealth setups.

### A definition of eHealth and mHealth

The World Health Organization (WHO) has defined Electronic Health (eHealth) as: “the cost-effective and secure use of information and communications technologies in support of health and health-related fields, including health-care services, health surveillance, health literature, health education, knowledge and research” [5]. Also, the WHO defines Mobile Health (mHealth) as: “mobile computing, medical sensor, and communications technologies for health care” [3].

### A brief description of the Israeli healthcare system

Israel’s health system is based on regulated competition among four non-profit health plans under the statutes derived from the 1995 National Health Insurance Law. Patients are free to choose annually among the four plans that are required to provide a standard basket of health services. Health plans are budgeted through risk adjusted capitation payments from government. Hospitals are financed based on services sold to the health plans and government subsidies. Health plans and hospitals have all developed their own health information systems, and recently government has intervened seeking to establish universal interoperability based on a system that will retrieve information about a patient from all the health plans. This information will be displayed only to the physician at the point of care and will disappear after the medical appointment is finished. Each of the health plans, as described below, has its own programs regarding patient access to EMRs (Electronic Medical Record) and the use of eHealth and mHealth.

### eHealth and mHealth in the Israeli agenda

Despite the fact that Israel has adopted a national information policy and a national ePolicy, it has not yet adopted a national eHealth policy, which is something the Ministry of Health (MOH) has been working towards in recent years. In Israel 100 % of the physicians at healthcare providers have access to their patients’ EMR. For purposes of comparison in the United States in 2013, according to data from the Centers for Disease Control and Prevention, only 78 % of office-based physicians used any type of EMR and 48 % of office-based physicians reported having a system that met the basic criteria of an EMR [6].

In recent years, not only was an EMR implemented in Israel, but also, the health plans have been providing different services on-line and some via mobile phones. With these services the health plans have granted the patients access to their own healthcare information from a computer or smartphone, in a user-friendly way and with clear information, a key step towards more complete patient empowerment [5]. This system is called Personal Health Record (PHR). Additionally, administrative services are offered via the PHR and other services such as electronic prescriptions or ePrescriptions began to be offered. At the onset of ICT implementation within the health plans the government exercised no defined role, neither as a regulator nor as a facilitator [7]. More recently, however, the administration of the MOH started to acknowledge the benefits of eHealth and mHealth and it has been working on national projects such as the National EMR that finally led to the Health Information Exchange (HIE) project [8]. This project is intended to facilitate the sharing of information at the point of care between different healthcare organizations.

### A brief description of the Portuguese healthcare system

Since 1979, the Portuguese health system is organized as a National Health Service (NHS). The local MOH owns and operates both hospitals and community based clinics. The private sector is also active in health delivery, and the government has sought to privatize some of its health delivery systems. Certain sectors of the population, such as civil servants, are covered by special insurance arrangements, and about 20 % of Portuguese citizens hold voluntary health insurance. In general, however, it is a tax funded system intended to provide equal access to all citizens free at the point of service [9].

### eHealth and mHealth in the Portuguese agenda

The introduction of ICT in health began in the 1990’s with the MOH designing a basic information system that would support the management and control of the flow of users, the standardization of clinical and administrative data, and the enabling of automatic billing and improvement in the communication between health providers [10, 11]. Different solutions were developed for hospitals, primary health centers, medical use and nurses respectively. Moreover, the MOH promoted the implementation of a unique system for privatized institutions, an example of tension between forces of decentralization on one hand, and centralized control of data on the other. Thus, while some private institutions rely on the government designed information systems, the fact that each institution had the freedom to choose the system to adopt without regulatory criteria to guarantee interoperability among them meant that some health delivery systems are isolated information wise. Thus, while the main driver of eHealth is the MOH, its reach falls short of the entire delivery system, a fact to be kept in mind when comparing to the Israeli case.

In Portugal, according to the National Statistics Institute, 93 % of the official’s hospitals^1^ and 73 % of the private hospitals have an EMR [12].

### An exploratory study of the role of ICT in two health systems

In the context of a European Union Funded Cost networking project on new developments in health systems management, part of the focus was on the role of patients, including user voice and empowerment. EHealth and mHealth were obvious related issues. It was noted, as indicated above, that initial research in Israel had demonstrated that increased deployment of ICT has, in the view of key stakeholders, increased patient empowerment and involvement [7]. Specifically, from the physician´s perspective, ICT has provided more information, though changes of these magnitudes were not easy in the beginning and good leadership was the key for success [13]. At the national level, it seems that the role of the government is becoming more important as shown by the involvement of it in a couple of national projects. However, some important barriers still need to be overcome, motivating further research on cost-effectiveness, privacy & data security issues and suitability of utilization by all kind of patients and physicians.

As the COST project encouraged Short Term Scientific Missions (STSM) for comparative research, it was decided to conduct a study comparing Israel to a country that appeared to have a somewhat different ICT evolution, namely, Portugal. It was envisioned that such a study could, at least in an exploratory way, help to understand how national initiatives and projects initiated by the organization could be integrated to advance a more patient-centered development of ICT in healthcare (Table 1). The STSM was accepted for presentation at the Medical Informatics Europe Conference that took place on May 27–29 in Madrid for which a shorter version of this paper was included in the conference proceedings [14].

**Table 1:** Comparison of Israel and Portugal

	Israel	Portugal
Demographic, Economic and Health Indicators^a^		
Population in millions (2012)	7,6	10,6
Area	20,770 km2	91,985 km2
GDP per capita (PPP) (2011)	27110	24440
Health Expenditure (% of GDP) 2011	7,7	10,4
Public Health Expenditure (2011)	61,5	64,1
Health Expenditure per capita (PPP int. $) (2011)	2171,9	2624,4
Life expectancy at birth (M/F)	79,9 / 83,6	77,6 / 84
No of physicians per 10,000 (2010)	36,5	38,7
No of beds per 1000 hab.	3,3	3,4
Infant mortality	3,5	3,1

^a^Source: Website of OECD

This table suggests similarities between the two populations. Nonetheless, we do not assume that the needs of the two populations imply that their ICT in health needs are similar. That is beyond the scope of this paper which will focus solely on the evolution of ICT in health in both countries. The degree to which the latter is affected by population needs is not addressed.

## Methods

### The qualitative approach

We used a qualitative, phenomenological approach seeking to reveal the perceptions of key health system actors regarding the implementation of eHealth and mHealth. At an early stage of the research on this subject, we decided to study a purposefully selected sample of people holding key positions within the health care system. These respondents could be expected to provide first-hand knowledge regarding the rolling out of ICT technologies in the health system, as well as their views regarding the impact on patients, including empowerment. Exploring patient empowerment was not so straightforward. While qualitative and quantitative study of patients and consumers was beyond the scope of this study, it did hold the potential to greatly inform such work, which has subsequently been initiated.

### Population of study and sampling

As mentioned, purposeful sampling was used to select study participants. The selection of participants was the opposite of theoretical random selection [15]. This matches the phenomenological design of the study in the spirit of Groenewald [16] who proffered that the phenomenon “dictates” the type of participants.

In qualitative studies, the size of the sample is not always clearly defined. Typically, data is gathered until the point of theoretical saturation, i.e., additional interviews are unlikely to provide new perspectives. Additionally, the researcher searches for participants who can provide rich and accurate information. Thus quality is more important than quantity (Table 2).

**Table 2:** Final sample of interviewees

Israel		Portugal	
**Stakeholders**	#	**Stakeholder**	#
**Healthcare Management Organization**	8		
• Evaluation and Planning (2)			
• Legal Department (2)			
• Director of Health Services			
• Director of Nurses			
• Family Doctors (2)			
**MOH**	3	**MOH**	2
• Director of the Division of Medical Informatics		• Chief Information Officer (SPMS-Director of Shared Services form Ministry of Health)	
• Director of Public Health			
• Chief Executive Officer		• Chairman of the Entidade Reguladora da Saúde	
Other healthcare management organizations	2		
• Chief Information Officer			
• Director of Evaluation, Planning and Research			
**Patient’ Organizations**	1	**Patient’ Organizations**	3
• Organization for the Patient’s Right		• CEO-Respira-Association for people with Chronic Obstructive Pulmonary Disease (COPD) (2)	
		• CEO-Portuguese Diabetes Association	
**Hospitals**	1		
• Chief Information Officer			
**Private companies**	1	**Private companies**	1
• Chief Executive Officer of a vendor		• Health Director of a vendor	
**Academy**	1	**Academy**	1

While the Israeli sample brought the research, conducted in the framework of a master thesis over more than a year, close to saturation, the Portuguese sample, due to time constraints, was perhaps too small, and is therefore more suggestive of perceptions that would emerge in a longer term study.

### Data collection

In-depth interviews are generally done face-to-face and allow the researcher to get detailed and direct information about the interviewee’s thoughts, personal feelings and experiences. This tool was useful to fulfill the objectives of the work. The duration of each interview was approximately between 45–60 min and was done at the work place of the interviewees. The language used in all the interviews was English. Despite the fact that all the interviewees spoke English, this was a limitation as they sometimes had difficulties expressing their ideas. Consent for (1) recording the interview, (2) publishing all the information and (3) mentioning the name and job position in the work was obtained from all the interviewees. Additionally, the interviewees were afforded the chance to mention something else or indicate if something should not be mentioned in the work.

A semi-structured questionnaire was used which granted a sense of general guidance but at the same time some degree of flexibility to introduce new questions as new topics came up in the conversation. Although most of the questions were asked to each interviewee, facilitating the comparison of answers, sometimes it was necessary to adapt the questions depending on the area covered with that specific key informant. For example, key sources from the government were asked more questions related to national projects, role of the government, and national policy, while questions directed at people in sick funds emphasized the implementation process within the organization.

Following from the background above, the questions were aimed at eliciting the views of respondents regarding very basic aspects of the development of an ICT in health policy and its implementation. We were looking for information on basic understandings of ICT, the motivations for its use in health, differences across sectors such as hospitals vs community care, and the role of government. The main questions tried to cover different topics like knowledge, experiences, examples, feelings and opinions. The questions were elaborated with the aim of capturing the personal perception about the phenomenon of adoption of technology in health. Main questions included:

1. What can you tell me about eHealth and mHealth? What do these terms mean to you? Which are the different tools in this area that you know?
2. What do you think about the reasons that motivate the implementation of these tools in the Israeli/Portuguese healthcare system and specifically in community healthcare?
3. What are the differences between the hospitals and the community health level when talking about eHealth?
4. In your opinion, what was the role of the government in the implementation? In which ways did the government support the implementation of the system?
5. If you have to map the different stakeholders in the implementation of eHealth tools, which ones would you include?
6. Which barriers did you see as important for implementing eHealth initiatives?
7. In terms of advantages and disadvantages, what is your opinion in relation to the situation before adoption of the technologies in terms of patient empowerment, control and decision and other relevant aspects/
8. What are the future trends?

When necessary, probing questions as *“Could you give me some examples?”* or follow-up questions like *“How did you solve that issue?”* were used to provide a more complete picture.

The interview protocol was used first in the Israeli study, and from the outset was found to be effective in getting respondents, who by and large answered with eagerness and at length, to reveal their points of view on the subject being studied.

In addition to the interviews (primary sources), secondary sources such as working papers, articles and presentations were analyzed both to discuss the results and to triangulate the findings.

### Analysis of data

All the interviews were recorded with a digital recording machine. Notes were taken during the sessions without the help of assistants and then the information was transcribed to a Microsoft Word processor. Analysis of the data was based on grounded theory [17] which consists of recreating and understanding the perspective of the interviewee by deriving categories and sub-categories found in the data. The creation of these categories and sub-categories, and the connection between them, helps to distill a holistic view of the object in analysis. Each word and sentence were then coded into categories that emerged from the texts.

## Results

### Importance of eHealth related with consumer voice

While in both Israel and Portugal, the interviewees mentioned that patient empowerment has increased, there were some disagreements, especially among patients’ organizations who expressed concerns regarding the risk of having patients get more information through Internet and Social Media. When interviewing the patients’ association in Portugal, the respondents mentioned that with some diseases the importance of eHealth tools is tremendous. Our respondents included one from an association related with respiratory diseases and another related with diabetes. In the first case, the interviewee mentioned that when the patient has a respiratory disease and weather conditions are not favorable, eHealth tools such as email, Internet, and mobile applications could be a great resource of communication, as in those cases it is very difficult to reach the hospital. Moreover, according to our respondents, ePrescriptions (electronic prescriptions) can help chronic disease patients get their prescriptions without going every time to visit their physician However, they also stress that face-to-face interaction is also important, especially to cope with patient anxiety. As one of them stated: *“people need from the physician*”.

Regarding the effect of the information found in the Internet on patient empowerment, the interviewees agreed that it is very important *“The physician can give you technical details, but is good to be informed and educated”* (Interviewee from a Portuguese patients’ association). Contrary to studies that suggest that patients may not get the right information [18] this interviewee feels that Internet accessed information would increase the effectiveness of discussions with patients.

*“diabetes is a part of life of people with diabetes and diabetes control reflects how people will live in the ….environment so we need to have proper implementation…people with diabetes they pay a difficult price to communicate with the healthcare team because people with diabetes they are their own managers of diabetes and in some point of their life they will communicate with the healthcare, they will go to consultation and they go to patient’s procedures but for 90 % of the time they would, they are managing the diabetes”* (Interviewee from a Portuguese patients’ association).

Also in Israel, interviewees highlighted the increasing role of the patients. Once they are aware of the possibilities and the barriers they can overcome, *“the patient is more educated, and can be really a partner in such a treatment, should be a partner”* (Member of the Israeli MOH), and should be involved.

Some of the respondents in the Israeli sample pointed to the risk that involved patients may not be able to discern between good and bad medical information found on the Internet. Another problem can be *too* much information:

*“Go to Google and try to ask a very simple question, I give you an example, what antibiotic I need to take for a specific infection, you will get in average between 6 to 7 million pages, you read the first two and that’s all, it is really a way to activate the patient?…”* (Interviewee from an Israeli private company)

Still, most respondents saw access to such information in a positive light. One of the physicians interviewed in Israel mentioned:

*“The educated consumer is our best customer. I don’t mind my patients knowing, having a look on the Internet to look up for their illnesses, if they are going to take responsibility of their health in that way I am for it”.*

It seems that the health plans need to balance the provision of care through eHealth with the personal care provided by the physician. “*You need to have balance, you need to balance, the need for care that simple physicians…will remain, this will not replace the physicians, would complement it…I have a problem now in providing care, we are providing care that it is sometimes ineffective that does not manage to get the patient involved enough and this could be an excellent tool for patient engagement…” (Interviewee from an Israeli health plan)*

Regarding the increasingly proactive role of patients, an interviewee from the Portuguese MOH mentioned that *“I think that patients are starting to have now a place they did not have”*, referring to the fact that in the last years the Ministry has started thinking about the patients as stakeholders. Interestingly, and somewhat differently from the Israeli perspective, this change is derived from use of ICT for administrative purposes. For example, the first initiative was “eBooking”, or the possibility to book an appointment with the general practitioners through Internet. Building on this, most of the important initiatives aimed at enhancing consumer voice were started. As an example, the MOH in Portugal has launched a Health Portal in 2008. This portal operates through a network involving all agencies reporting to the MOH, with a central core consisting of the General Secretariat of the MOH and the Central Administration of the Health System. All citizens can access and use it for free. It is intended to be a gateway that provides direct access to up to date information on health issues, as well as online services, news, health information and information regarding the institutional organization of the sector. It also offers search services about several health topics and other information on services offered by hospitals and health centers, such as their schedules, and pharmacy hours.

*“This means, of course, that they now contact the help line, they criticize, and they say this is not correct in my information; I think that if you have a button for… I think this kind of disease, you know, I cannot record my disease, you don’t have it in the drop list…I think we did not have a reason to meet patients’ associations and things like that…now they interact in the portal directly or we want them to do so, so, they will have an opinion, and they will have an opinion, and the project is growing, and I think should grow faster, and next year we will have to invest in that…”* (Interviewee from the Portuguese MOH).

Some of the patient’s associations will be plugged to the portal and information will be available. *“I think that increased a lot the transparency of the information”,* he added. According to the interviewee this portal is an important communication channel between the patients and the healthcare system.

Similarly, in Israel the relationship between ICT and patient role and satisfaction arose. From the interviews it was not clear if the patients need to be in the first stage of every implementation program in eHealth, but they are an important part of the evaluation of those programs, “*we have a lot of input from the patients”* said a key manager from one of the Israeli health plans, talking about satisfaction surveys done in the sick fund.

As mentioned by another key informant from another health plan *"by definition, you have to click in”*. The main areas of patient involvement were communication and feedback (from periodical surveys), social networks and patient education.

*“gradually, the attitude in the world is patient empowerment, this is the attitude, health is so expensive, without the support of the patient we cannot achieve anything, patient empowerment is a very important value, now we are communicating with patients through the Internet, no other way, this is the world”* (Interviewee from an Israel research institute)

### Impact of eHealth on physicians and medical organizations

When asked how the patient-physician relationship has changed, one of the interviewees in Portugal said that patients know more now as a result of Internet browsing and this shift in access to information can influence patient confidence in physicians,. The latter need to be prepared: *“That brings a huge responsibility to healthcare professionals because they have to know that patients look for that information”* (Interviewee from a Portuguese patients’ association).

Regarding issues like adaptation and change of paradigms of physician interaction with new technologies, results in both countries were similar, highlighting physicians’ reluctance.

*“…also the caregivers should feel comfortable with the technology; when we started to implement medical records you know, computerize medical records, the physicians had a hard time, you know, they have to talk, look to the patients, talk to the patient, feel the patient-now they have to press keys…”* (Interviewee from an Israeli health plan)

*“They get used to that, physicians always they, they are slow to accept changes, so every change is difficult for them. … there are also, I think there some good points as when they say what will happen with such a technology… for example I saw some concerns about patients getting to the data so they say, how they are going to understand the data, now they will have many, many questions and this bothers all the physicians, and so on, so there are many, many questions”* (Interviewee from the Israeli MOH).

These barriers were overcome as physicians began to understand the benefits of the tools and with the arrival of a new generation of physicians. This view was shared by the Portuguese interviewees. One of the interviewees in Portugal mentioned that it is important to share the ownership of innovation with the physicians as they may be reluctant to change: *“…you have to bring physicians to innovation, and sometimes they resist, sometimes they don’t like, but sometimes they don’t like because they are used, they are not in the center of innovation, sometimes you bring innovation to tell them what to do, instead of using them to tell how innovation should be done, so there is also this kind of gap.”* (Interviewee from a private Portuguese technology company).

*“It was variable, as expected it could depend on the specialties, could depend on the age of the physician, the interest in technology also and it depends also what was in written form before, and the we try to reproduce exactly what was written before”* (Interviewee from a Portuguese patient’s association).

According to the interviewee from the Portuguese diabetes patients’ association, during the first two years of implementation the association used the two systems, paper and electronic, but in the end the move to the electronic was mandatory for all physicians. The main barrier was that physicians were not used to technology, however the association motivated its physicians by showing the importance of sharing information and providing clinical information.

Physicians’ behavior can be affected by eHealth tools by changing the way decisions are made. For one of the interviewees, one of the most important advantages of eHealth tools is that it improves the way medical decisions are made by physicians as they have access to more information in real time about their patients, reducing the risk of malpractice.

From the side of the organization, eHealth tools can be a solution to the increasing costs the healthcare system faces today: *“eHealth for me is how to enable technology to help the healthcare system to build this new reality where they can provide better care at a lower cost”* mentioned the interviewee from a technology company located in Lisbon.

### Perceptions about the government

As other studies revealed, all the interviewees agreed that the Portuguese government has an important role in the implementation of eHealth services and its development [4]. According to the interviews, most of the software implemented in the hospital and healthcare services community was developed by the government which facilitates the interoperability between some of the systems.

One of the interviewees mentioned: *“The Portuguese health system is based on a National Health Service (NHS). This NHS is the main provider and the main funder of health care. Thus, the implementation of eHealth on a nationwide scale is highly dependent on the government.”*

Apparently, according to one of the interviewees, the role of the government could be divided into different stages but the tradition was that the Portuguese MOH was generally the developer. Then in 2000 there was a strong debate about the role of the government, including the idea that government should focus more on regulation and outsource the development of software [4]. In the end, mainly due to economies of scale and the high level of dependency of hospitals on the software created by the MOH, it was decided to build in house solutions.

*“[interoperability] is easier… the risk there, have to accept the risk, is that you will have such a big thing taking care of so many things, that if it doesn´t move, most of the system doesn’t move”* (Interviewee from the Portuguese MOH).

In addition, other interviewees observed that government intervention may slow down innovation, *“to be honest if this only depends on the politicians, they come and go every four years, and every time they stop everything, people in the field, should be, should be a lot more involved and also be responsible for keeping the trend…”* (Interviewee from a Portuguese private company)., The interviewee also added that one of the roles that the public system should have is to support and facilitate the investments of the private sector, and moreover, that there should be more cooperation between the private and public sector in order to not slowdown the pace of innovation for better patient care. (Table 3).

**Table 3:** Comparison of Israel and Portugal-eHealth in the country

	Israel	Portugal
**ICT development indicators** ^a^		
ICT Development Index	6.19	5.77
ICT Development Index rank	27	32
Cellular subscribers per 100 hab (2011)	122	115
**eHealth Policies** ^a^		
National eGovernment Policy	Yes (2004)	Yes (before 2000)
National eHealth Policy	Yes (in process)	Yes (2008)
Regulation on eHealth	Yes	Yes
National telemedicine policy	No	Yes
mHealth initiatives are conducted in the country	Yes	Yes
Formal evaluation and/or publication of mHealth initiatives	No	No
**Results from the research**		
Time of development of eHealth initiatives	1985	1990
Starter of initiatives	Health plans (bottom-up)	Government (top-down)
Innovation	Innovation culture, start-up nation	Slow innovation in the area
Interoperability	Yes (since 2014)	Yes (since 2007)
Health Portal	Yes	Yes

^a^Source: WHO [27]

In Israel the story was different. The implementation of eHealth tools has typically been an initiative of the health plans, rather than the government. This is a consequence of the Israeli management and innovation culture. After the healthcare system reform law of 1995, healthcare providers *“understood that without data, they will be lost, they will lose in the competition with the other health plans, and this is a management aspect and also can be used very efficiently for quality improvement like the different… the quality improvement indicators that we are using”* (Interviewee from a Israeli health plan).

*“…the current government and MOH decided to work differently, ´we should continue including what is already in place. This is a different approach to that in many places in the world, in the world, this is how it works, in many places when they try to dictate everything, like England, the UK, after 20 billion pounds already, and it has failed miserably” (Interviewee from an Israeli private company).*

The health plans were key players in developing the different information systems in Israel. *“There was no involvement of the MOH in all of what we did to set up our information system”,* mentioned a key informant from one of the health plans. *“There is a mixed blessing here that they let us do what we need to do and they don’t …(get) in our way … that’s an advantage but on the other hand there are a lot of issues that have to be addressed and should be addressed nationally”*, she added. But when asked about a more active government role, the answers from the interviewees were mixed. The roles attributed to the government ranged from watchdog/regulator to a more policy-oriented role in dealing with issues from a national perspective, such as adoption of a national eHealth strategy, or economic and ethical aspects of eHealth tools.

Another issue is the topic of interoperability, meaning the capability of sharing information between different organizations. In this sense, one key informant from another healthcare organization mentioned *“Sick funds had to do it by themselves and they did it quite well I think locally, and because we couldn’t not advance towards the integration, that I believe is strongly necessary, the government is now stepping in and it’s playing an important role, even without providing the large sums of money and maybe even by taking credit for what is happening ….this is a very good thing, and it’s good for everyone”* (Interviewee from an Israeli health plan).

### Barriers and challenges

Answers about barriers and challenges vary between the interviewees. One representative of the Portuguese MOH mentioned that one of the most important barriers and challenges is the expectation from people that eHealth effects are immediate. He mentioned that this is an important issue as most of the eHealth initiatives could take time to be fully implemented.

When talking about costs, interviewees did not agree. While some of them thought that high costs and unknown cost-effectiveness may be a barrier, others mentioned that eHealth initiatives do not necessarily need to be cost effective but simply effective. *“Nobody asks you if your bank account was cost-effective”* (Interviewee from the Portuguese MOH). In other words, the deployment of eHealth and mHealth should not be slowed down by premature demands for proof of cost effectiveness.

Lack of leadership seems to be important for some of the interviewees as one of the barriers for implementation: *“… action does not result from speeches”* (Intverviewee from a Portuguese private company).

Another barrier mentioned by one of the members of a Portuguese patient association was the fact that in some cases patients may feel that they do not receive anything in return for their input and for their information. The interviewee mentioned the importance of two-way information (Table 4).

**Table 4:** Comparison of Israel and Portugal-main barriers and challenges (according to the interviewees)

	Israel	Portugal
Lack of knowledge of applications	No	No
Cost effectiveness unknown	No	No
Lack of legal policies/regulation	Yes	-
Perceived costs too high	No	Yes/No
Underdeveloped infrastructure	No	No
Lack of research	Yes	-
Lack of leadership	-	Yes
Physicians	Yes	Yes

In Israel, interviewees mentioned the lack of regulation, the lack of research, and physicians as the main the barriers. Technology and costs did not seem to be barriers to overcome for them, similar to their Portuguese counterparts.

Regarding the lack of regulation, as mentioned by one of the interviewees, technology is moving so fast that it is very difficult for the regulatory system in Israel to move along at the same pace.

*“Confidentiality, this is the major concern for the citizens of this country and then you understand that efforts to collect all the medical data about someone can be used in order to take good care of him but can be used by people that do not really want to take care of him, to know things about him, or you know to insurance companies, different intelligence agencies and so on, it’s something that should be restricted and I believe that the national project is taking good care of this”* (Interviewee from an Israeli health plan).

## Discussion

### Increasing consumer voice through eHealth and changing physicians behaviour

Thanks to the development of new information and communication technologies in health, patient empowerment and consumer voice has certainly increased, as patients have access to more information in real time, anywhere/anytime, and thus have more capacity to take decisions and are better informed. Moreover this increased access allows them to be more active in taking care of their health and be part of a patient-centered medicine.

According to the International Alliance of Patient’s Organizations (IAPO) there is more than one definition of patient-centered healthcare, but we have taken a definition that fits our interests: *“Being patient centered actually means taking into account the patient’s desire for information and for sharing decision making and responding appropriately, allowing the patient the opportunity to decide how much involvement and responsibility they want”* [19].

The idea of patient empowerment is one of the objectives of the implementation of ICT technologies in health. According to the WHO [20], empowerment of health is defined as: *“…a process through which people gain greater control over decisions and actions affecting their life”.* To change the role of the patient from passive to active, to make him aware of his own health, the patient needs information as well as knowledge about how to use this information. According to a document from the WHO [21], *“Patients can be empowered only after having gathered enough information, understand how to use the information, and are convinced that this knowledge gives them the opportunity, and the right, to participate in helping to keep health care safe while not deflecting the responsibility away from their health-care workers”.*

Based on this definition, ICT in health as implemented today can help improve the quality of decisions made by patients, reduces the asymmetry of information between patient and physician in some way and provides patients with a greater sense of control. However, various barriers exist, such as the change in the interaction between physician and patient, and information overflow.

From the findings of the interviews, it seems that this increasing access to information is not a risk and that having a more educated patient is seen as an advantage. While in Israel, some concerns about patient eHealth literacy, the “too much information” syndrome, and the risks of the security and uncertainty of what is presented as evidence based information were raised by some of the interviewees, this was not the case in Portugal. The possibility to discuss information and previous knowledge with the physicians was seen as something desirable. As one of the interviewees emphasized, the physician should give the technical details, but the knowledge can be acquired by the patient himself. It is important to remember that the notion of patient empowerment is delicate and still evolving and depends on patient attitudes and socio-economic factors that our respondents did not relate to, speaking about users in general and not relating to different levels of knowledge, socioeconomic factors, and cultural groups.

Due to the large amount of mobile applications developed without widely accepted scientific data, patients can download thousands of applications to their mobile phone to control their health, but the lack of research and validity of these apps pose a risk, followed by the question, who is accountable?

One of the common aspects found was the fact that while eHealth tools can facilitate access to information without visiting the physician, the patient-physician relationship remains important. In addition, in both countries physicians are an important barrier for implementation. Medical schools are often conservative, and physician adaptation to new tools and utilization of new mechanisms of communication with patients are aspects that organizations and government should take into account. As mentioned [2], “many IT applications require the forging of new relationships between clinicians and institutional providers, which may be slow to develop. For example, some have observed that the deeply ingrained economic distrust and cultural conflict between physicians and hospitals has impeded the adoption of IT applications that requires web-based integration [22]”. Other authors refer to physicians’ reluctance in adopt ICT systems in their daily routine due to criticisms regarding the quality and lack of innovativeness of some IT products [23, 22, 2, 24, 4].

### Policy implications

As patients are becoming more involved through the utilization of different tools as EMR, PHR, mobile apps and other communication channels, regulation and research need to keep pace. With more empowerment comes more responsibility for making decisions. However, the decision making should be based on true evidence-based information.

In terms of physician behavior and organizational change, more education for the healthcare workforce should be integrated in any plan. Integrating an orientation to ICT in the formation of medical providers needs to be done without leading to deterioration of the patient-physician relationship, and without jeopardizing patients’ safety and the quality of clinical decisions. As described by Espanha [25], patients are more informed and thus the level of autonomy has increased. These changes pose a challenge to the physicians and health institutions that may need to re-think the physician/patient relationship.

### The role of the government and a national eHealth strategy

The Portuguese case presents differences from the Israel situation in the balance of top down vs bottom up initiatives. While in Israel innovation came from the field at earlier stages (around 1985–1990) and government involvement came later (bottom-up strategy), in Portugal the government involvement can be found earlier on in the process of implementation. In a document published by the European Union, Da Costa Pereria et al. [26] mention that national policies started to be drafted by 2007 when a consulting firm presented the first document which describes governance, action plans and budgets. In 2008, the government presented the Plano Tecnológico da Saúde (PTS) which describes the policy approach to eHealth. Finally, in 2009–2010, the Central Administration of the Health System (Administração Central do Sistema de Saúde - ACSS) published a policy paper about a national EMR. This document had the advantage that it was published after a series of discussions between 30 different stakeholders including government, hospitals, primary care, professionals and university. This process shows that eHealth initiatives came from governmental directives with few initiatives spurred locally. The authors of the document mentioned above indicate that the reason is in part because of (very much opposed to the situation in Israel) lack of management and leadership, as well as the level of interest in the eHealth domain at the local level. This could be also due to structural differences between the Portuguese NHS, characterized by a strong presence of government in health delivery organizations, as opposed to the Israeli structure of autonomous health plans.

This difference has important consequences. In Israel, maybe because of cultural aspects, each organization developed its own system without any kind of coordination with the other. The result is that systems were not interoperable and future sharing of information presents a challenge, which the current government is facing. In Portugal, while there is also a problem of interoperability due to the concomitance of private and public influence in this area, with the government being one of the software builders, interoperability is less of an issue, but the pace of innovation is slower.

Finally, and as mentioned above, we have not taken into account differences in population needs that might in some way influence the evolutionary pattern of ICT in health in both countries.

### Policy implications

National strategies seem to be important to coordinate the different stakeholders in the system and to reach interoperability. Strategies need to facilitate innovation at the same time that regulation needs to move at the same pace of technological developments. In addition, in order to create a holistic strategy, all stakeholders, including patients, physicians, organizations, private sector and academy need to be included. Espanha [25] mentions that “…the health professionals play a central role in the constant (re)definition of the position of ICT in the health system. To know them is extremely important for (re)defining the role of ICT in the health system”.

## Conclusions

Healthcare systems worldwide are facing the same challenges: increasing costs, shortage of human resources, aging of the population and the prevalence of chronic diseases. In order to find a solution, the healthcare sector has incorporated different types of ICT, with the intention to provide healthcare at a lower cost without reducing the safety of patients.

The incorporation of ICT in healthcare appears to have increased patient empowerment and has activated the patients, making them more aware of their healthcare and more responsible, by giving more access to information. It has also challenged the patient-physician relationship and both sides will have to adapt.

Both the Israeli and Portuguese healthcare systems are part of this trend. This research suggests that both patient empowerment and physician behavior has changed. Based on interviews with key informants, in both countries, ICT has increased access to information allowing the patients to make better (or at least more informed) decisions and to be more active in their care. In both countries, the provider/patient relationship has been changing in ways that call for concerted attention to prevent deterioration.

In terms of government involvement, the comparison of the top down Portuguese and bottom up Israeli evolution of ICT is suggestive. Both strategies have their advantages and disadvantages. A top-down strategy facilitates the interoperability of systems and the exchange of information as all healthcare providers use the same or at least similar system. However, it depends on the government direction, priorities, and bureaucracy. On the other hand, a bottom-up strategy accelerates innovation as competition for being the most innovative is high, but when trying to share information, interoperability is a challenge.

The development of eHealth and mHealth tools is an increasing trend. Patients probably will be more aware of their health and will use different technologies to assess their data and information by themselves. The professional formation of medical providers will need to adapt to this new paradigm of a more activated patient, avoiding reductions in the quality of care. A national strategy could be helpful in solving these challenges.

## Endnote

^1^Official Hospitals are defined as those that are protected administratively by the state, regardless of ownership of the facilities. It can be: Public-under the Ministry of Health or Regional Health Departments, whose access is universal; Military-under the Ministry of National Defense; Paramilitary-under the Ministry of Internal Affairs; Prison-protected by the Ministry of Justice.
